# Quantitative changes in platelet count in response to different pathogens: an analysis of patients with sepsis in both retrospective and prospective cohorts

**DOI:** 10.1080/07853890.2024.2405073

**Published:** 2024-09-20

**Authors:** Shao Hua Fan, Ming Min Pang, Min Si, Chong Feng Cao, Mei Chen Yan, Yue Xu, Ting Yu Meng, Mei Yin, Hao Wang

**Affiliations:** a Department of Critical Care Medicine, Central Hospital affiliated to Shandong First Medical University, Jinan Central Hospital affiliated to Shandong University, Jinan, China; b Department of Critical Care Medicine, Qilu Hospital of Shandong University, Jinan, China; c Shandong Key Laboratory of Cardiovascular Proteomics and Department of Geriatric Medicine, Qilu Hospital, Cheeloo College of Medicine, Shandong University, Jinan, China

**Keywords:** Sepsis, microorganism, platelet count, thrombocytopenia

## Abstract

**Background:**

Thrombocytopenia is commonly observed in patients with sepsis and is an independent risk factor for poor prognosis. However, the changes of platelet count caused by different pathogens can vary significantly. Our study aims to evaluate the quantitative changes in platelet count in response to various pathogens.

**Material and Methods:**

We retrospectively analysed data of 3044 patients with sepsis from Medical Information Mart for Intensive Care (MIMIC, 2008–2019) database and prospectively collected data of 364 patients with sepsis from our local cohort of the Shandong Bloodstream Infection and Sepsis Collaboration Study (SBISC, 2020–2022). Propensity score matching (PSM) was employed to control for baseline differences in variables, except for the causative pathogen.

**Results:**

Multivariate logistic analyses of both original and PSM populations identified *Candida*, *Escherichia*, *Klebsiella*, and *Serratia* species posing a higher risk for thrombocytopenia compared to others. Restricted cubic spline (RCS) curves showed L- or U-shaped associations between platelet count and 28-mortality with various cut-off values among different pathogens: ranging from 96 × 10^9^/L in *Candida* species − 190 × 10^9^/L in *Klebsiella* species.

**Conclusion:**

Our present findings indicate a pathogen-specific effect on platelet count, highlighting the importance of monitoring thrombocytopenia in patients infected with above microorganisms. Clinicians need to consider pathogen-specific thresholds when intervene on platelet count.

## KEY MESSAGES

This study validated the differential incidence of thrombocytopenia among various pathogens within two distinct populations.

*Candida*, *Escherichia*, *Klebsiella*, and *Serratia* species were identified as having a notably higher risk of causing thrombocytopenia compared to other pathogens.

We observed L- or U-shaped relationships between platelet counts and 28-day mortality in *Candida* species*, Enterococcus* species*, Escherichia* species*, Enterobacter* species*, Staphylococcus* species, and *Klebsiella* species with platelet count cutoff values of 96 × 10^9^/L, 100 × 10^9^/L, 100 × 10^9^/L, 146 × 10^9^/L, 152 × 10^9^/L, and 190 × 10^9^/L, respectively.

## Introduction

Platelets are one of the principal components involved in hemostasis and play a crucial role in the host’s immune responses activated during infection. Notably, the response of platelets to bacterial or fungal infections has received particular attention [1–3]. During infection, platelets enhance the immune response through several mechanisms, including inflammatory reactions against the infection [4, 5]. However, these inflammatory pathways, in turn, may lead to the elimination of activated platelets and platelets interacting with pathogens. Platelets possess the ability to identify and bind directly or indirectly to different pathogens through a diverse range of receptors; moreover, pathogen-secreted products may modulate platelet function and promote platelet adhesion or aggregation [3]. However, the systematic activation of platelets by pathogens can result in significant platelet consumption and potentially lead to their destruction [2, 3].

Thrombocytopenia is defined as a platelet count of <150,000/μl and is typically observed during severe infection [6]. In general, thrombocytopenia occurs in 20–58% of septic patients [7]. Extensive research has shown that the development of thrombocytopenia is an independent risk factor for the poor prognosis of critical infection [6, 8–10]. Given the diverse and complex mechanisms through which different pathogens interact with platelets, the platelet response to specific pathogens can vary during infection, potentially leading to pathogen-specific changes in platelet counts. However, up to now, few studies have investigated the potential relationship between specific pathogens and the occurrence of thrombocytopenia, and the critical thresholds for platelet counts requiring clinical intervention remain unclear. Determining the pathogen-specific platelet changes in large cohorts with a sufficient number of microbial species is necessary to determine the causes of thrombocytopenia and the appropriate clinical interventions in patients with sepsis with different pathogens. Our study aims to bridge these research gaps by evaluating the quantitative platelet response to different pathogens in patients with sepsis, and so as to provide reference for appropriate intervention.

## Materials and methods

### Study cohort and data sources

Patients with sepsis infected with various pathogens confirmed by blood cultures, were identified from the Medical Information Mart for Intensive Care (MIMIC, 2001-2019) database and our local multi-center prospective observational cohort from the Shandong Bloodstream Infection and Sepsis Collaboration study (SBISC, 2020-2022). The MIMIC database, a public, open database managed by the Massachusetts Institute of Technology (MIT) and Beth Israel Deaconess Medical Center (BIDMC), contains extensive data collected from patients admitted to BIDMC [11, 12]. Use of the MIMIC database for research purposes has been approved by the Institutional Review Boards of BIDMC and MIT, and the informed consent of this study was waived. For our local multi-center prospective observational cohort, the protocol has been approved by the Ethics Committee of Qilu Hospital, Shandong University (approval number KYLL-2018153). The participants or their guardians in this study have provided written informed consent for the use of their data, which has been anonymized without distorting the scholarly meaning.

### Study population

We included consecutive sepsis 3.0 patients (aged ≥16 years) with specific causative pathogen. The inclusion criteria were (1) the causative pathogen was confirmed by blood cultures; (2) survival time >48 h. Patients were excluded according to the following exclusion criteria: (1) potential contamination of the microbial culture with other microorganisms according to previous studies [13, 14] or identification of more than one pathogen; (2) presence of other conditions that may cause thrombocytopenia or pre-existing thrombocytopenia, including hematological system diseases (hemolytic uremic syndrome, thrombotic thrombocytopenic purpura, immune thrombocytopenia, disseminated intravascular coagulation [DIC], hematologic malignancy, etc.), severe liver disease (including liver cirrhosis, significant hepatic impairment, portal hypertension, and esophageal varices), and acquired immune deficiency syndrome (AIDS), or drug-indued thrombocytopenia, as well as radiotherapy or chemotherapy within the preceding 3 months; (3) Severe heart failure or end-stage chronic renal failure; and (4) more than 20% missing values. For patients with multiple episodes of microbiology culture, only data from the first episode were retrieved.

### Variables

We collected patients’ baseline data (age, gender, height, weight, medical conditions), vital signs and extreme laboratory outcomes (the highest or lowest values, as appropriate) during the onset of sepsis. Acute severity scores, namely, Simplified Acute Physiology Score II (SAPS II)/Acute Physiology and Chronic Health Evaluation II (APACHE II) and Sequential Organ Failure Assessment (SOFA) scores, were also collected, along with data on life support treatment. The codes used for MIMIC data extraction are available at Github (https://github.com/MIT-LCP/mimic-code.git). Medical conditions of MIMIC patients were identified and extracted according to the ICD-9 and ICD-10 coding system.

### Definitions

Thrombocytopenia was defined as circulating platelet count of <100 × 10^9^/L according to a previous study [6]. The nadir platelet count was defined as the lowest platelet count during the first 2 days after sepsis onset. Septic shock was defined according to the Sepsis-3 criteria [15].

### Outcomes

The primary outcome was the incidence of thrombocytopenia in various pathogens. The secondary outcome was 28-day all-cause mortality.

### Statistical analysis

All data were analysed using SPSS 26.0 and R 3.5.3. Categorical data are shown as numbers and frequencies, continuous parametric data are shown as mean and standard deviation (SD), continuous nonparametric data are shown as median and interquartile range (IQR). Categorical data were analysed using chi-square test. Continuous parametric data were analysed used one-way ANOVA or Student’s *t*-test. Continuous nonparametric data were analysed using Wilcoxon rank-sum test. *p* < 0.05 (two-sided) was considered to indicate statistically significant differences. The proportion of patients with thrombocytopenia was calculated for different microorganisms separately. We used multivariable logistic regression models to assess for the independent effect of different pathogens on the circulating platelet count. As thrombocytopenia during the infectious period may be caused by other factors, we used propensity score matching (PSM) to reduce mismatch among groups infected by different pathogens and determine the association between specific pathogens and thrombocytopenia. Kaplan-Meier curves were used to create survival plots for stratifying patients on the basis of platelet count. Multivariate Cox proportional hazards models were used to analyse the relationship between thrombocytopenia and 28-day mortality. Restricted cubic spline (RCS) based on multivariable Cox proportional hazard models using likelihood ratio test was applied to evaluate the association between platelet count and 28-day all-cause mortality. Detailed information regarding PSM can be found in eMethods in Supplementary material 1.

## Results

### Patient characteristics

**Figure 1.**
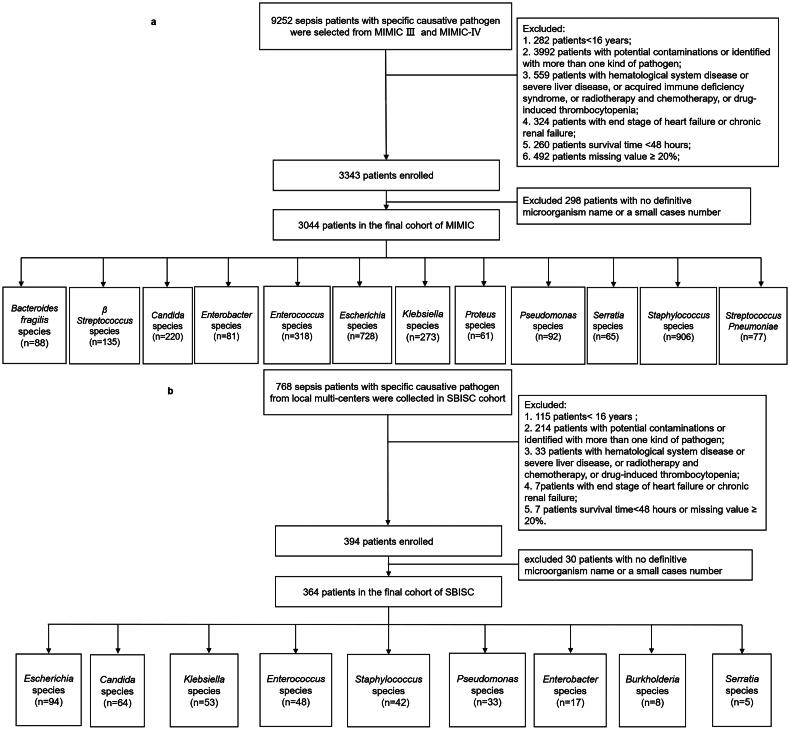
Study cohort. (a) The process of included and excluded patients with sepsis in MIMIC cohort. (b) The process of included and excluded patients with sepsis in SBISC cohort.

From the MIMIC-III and MIMIC-IV database, we extracted data for 9252 septic patients with specific causative pathogen: 6208 patients were excluded according to the inclusion and exclusion criteria, and 3044 patients were analysed in the final cohort (Figure 1a). The three most common bacteria were *Staphylococcus* species (906/3044), *Escherichia* species (728/3044), and *Enterococcus* species (318/3044). Among the 3044 patients, 838 patients had thrombocytopenia (platelet count, ≤100 × 10^9^/L) and 2206 patients did not have thrombocytopenia (platelet count, ≥100 × 10^9^/L). Basic characteristics, clinical data, and disease severity according to platelet count levels are shown in Table 1.

**Table 1.** Patient characteristic in MIMIC cohort.

Variables	Total *n* = 3044	PLT < 100 × 10^9^/L *n* = 838	PLT ≥ 100 × 10^9^/L *n* = 2206	*P*
Age, median [IOR]	68.6 [56.5;79.3]	67.0 [55.1;78.3]	69.2 [56.9;79.6]	0.002
**Gender**				0.710
Male, n (%)	1718 (56.4)	478 (57.0)	1240 (56.2)	
Female, n (%)	1326 (43.6)	360 (43.0)	966 (43.8)	
**Medical conditions**				
Myocardial infarct, n (%)	458 (15.0)	139 (16.6)	319 (14.5)	0.159
Congestive heart failure, n (%)	961 (31.6)	258 (30.8)	703 (31.9)	0.597
Chronic pulmonary disease, n (%)	727 (23.9)	188 (22.4)	539 (24.4)	0.268
Mild liver disease, n (%)	414 (13.6)	198 (23.6)	216 (9.79)	<0.001
Diabetes, n (%)	958 (31.5)	232 (27.7)	726 (32.9)	0.006
Renal disease, n (%)	541 (17.8)	132 (15.8)	409 (18.5)	0.108
Malignant cancer, n (%)	373 (12.3)	127 (15.2)	246 (11.2)	0.003
**CCI, median [IQR]**	5 [4;7]	5 [4;7]	5 [4;7]	0.144
**Primary infection source**				
Lung, n (%)	1077 (35.4)	316 (37.7)	761 (34.5)	0.107
Abdomen, n (%)	1089 (35.8)	328 (39.1)	761 (34.5)	0.019
Urinary, n (%)	641 (21.1)	184 (22.0)	457 (20.7)	0.484
Skin and soft tissue, n (%)	128 (4.20)	43 (5.13)	85 (3.85)	0.142
Central nervous system, n (%)	52 (1.71)	13 (1.55)	39 (1.77)	0.798
Endocardium, n (%)	207 (6.80)	66 (7.88)	141 (6.39)	0.170
Intravascular devices, n (%)	332 (10.9)	79 (9.43)	253 (11.5)	0.121
other, n (%)	130 (4.0)	30 (3.1)	100 (4.3)	0.116
Unknown, n (%)	242 (7.95)	70 (8.35)	172 (7.80)	0.666
**Lab value (median [IQR])**				
WBC, ×10^9^/L	12.8 [8.6;19.4]	9.9 [6.8;17.6]	13.7 [9.1;20]	<0.001
PT, second	15.9[14.1;20.2]	17.6[15.1;23.5]	15.4[13.9;18.9]	<0.001
INR	1.4 [1.2,1.8]	1.5 [1.3,2.0]	1.3 [1.2,1.7]	<0.001
APTT, second	37.5 [30.7;59.1]	43.6[33.6;70.5]	35.7 [29.9;53.0]	<0.001
Bilirubin, mg/dl	1.0 [0.5;2.3]	1.8 [0.9;4.3]	0.8 [0.5;1.6]	<0.001
Creatinine, mg/dl	1.5 [1.0;2.3]	1.7 [1.2;2.9]	1.3 [0.9;2.1]	<0.001
Lactate, mmol/L	2.1 [1.4;3.3]	2.8 [1.9;4.9]	1.9 [1.3;2.9]	<0.001
**Microorganism**				0.003
*Bacteroides Fragilis* species, n (%)	88 (2.89)	18 (2.1)	70 (3.2)	
*β Streptococcus* species, n (%)	135 (4.43)	38 (4.5)	97 (4.4)	
*Candida* species, n (%)	220 (7.23)	76 (9.1)	144 (6.5)	
*Enterobacter* species, n (%)	81 (2.66)	27 (3.2)	54 (2.4)	
*Enterococcus* species, n (%)	318 (10.45)	65 (7.8)	253 (11.5)	
*Escherichia* species, n (%)	728 (23.91)	224 (26.7)	504 (22.8)	
*Klebsiella* species, n (%)	273 (8.97)	80 (9.5)	193 (8.7)	
*Proteus* species, n (%)	61 (2.00)	15 (1.8)	46 (2.1)	
*Pseudomonas* species, n (%)	92 (3.02)	26 (3.1)	66 (3.0)	
*Serratia* species, n (%)	65 (2.13)	23 (2.7)	42 (1.9)	
*Staphylococcus* species, n (%)	906 (29.76)	231 (27.6)	675 (30.6)	
*Streptococcus Pneumoniae*, n (%)	77 (2.53)	15 (1.8)	62 (2.8)	
**SAPS II, median [IQR]**	38 [30;49]	42[34;54]	37[29;47]	<0.001
**SOFA score, median [IQR]**	7 [5,10]	10 [7,13]	6 [4,8]	<0.001
Septic Shock, n (%)	2032 (66.8)	654 (78.0)	1378 (62.5)	<0.001
Vasopressor usage, n (%)	1466 (48.2)	553 (66.0)	913 (41.4)	<0.001
Mechanical Ventilation usage, n (%)	1404 (46.1)	482 (57.5)	922 (41.8)	<0.001
Extra-corporeal epuration, n (%)	180 (5.91)	54 (6.44)	126 (5.71)	0.497
**Anti-platelet drugs**	958(31.5)	204 (24.3)	754 (34.2)	<0.001
**Mortality**				
28-day death, n (%)	517 (17.0)	225 (26.8)	292 (13.2)	<0.001
In hospital death, n (%)	487 (16.0)	227 (27.1)	260 (11.8)	<0.001

Date expressed as median(Q1-Q3) or number (percentage). *PLT*, Platelet; *CCI*, Charlson comorbidity index; *WBC*, White blood cells; *PT*, prothrombin time; *INR*, International normalized ratio; *APTT*, activated partial thromboplatin time; *SAPS*, Simplified Acute Physiology Score; *SOFA*, Sequential Organ Failure Assessment score.

From the SBISC cohort, we recruited 768 consecutive septic patients with specific causative pathogen: 404 patients were excluded according to the study criteria, and 364 patients were included in the final analysis cohort (Figure 1b). The three most common pathogens were *Escherichia* species (94/364), *Candida* species (64/364), and *Klebsiella* species (53/364). Among 364 patients, 116 had thrombocytopenia (platelet count, ≤100 × 10^9^/L) and 248 patients did not have thrombocytopenia (platelet count, ≥100 × 10^9^/L). Basic characteristics, clinical data, and disease severity according to platelet count levels are shown in Table 2.

**Table 2.** Patient characteristic in SBISC cohort.

Variables	Total *n* = 364	PLT < 100 × 10^9^/L *n* = 116	PLT ≥ 100 × 10^9^/L *n* = 248	*P*
Age, Median [IQR]	60.0 [45.0;73.0]	59.0 [42.5;74.0]	60.5 [46.0;73.0]	0.783
**Gender**				0.945
Male, n (%)	236 (64.8)	76 (65.5)	160 (64.5)	
Female, n (%)	129 (35.2)	41 (34.5)	88 (35.5)	
**Medical conditions**				
Myocardial infarct, n (%)	17 (4.67)	4 (3.45)	13 (5.24)	0.625
Congestive heart failure, n (%)	157 (43.1)	44 (37.9)	113 (45.6)	0.209
Chronic pulmonary disease, n (%)	44 (12.1)	18 (15.5)	26 (10.5)	0.179
Mild liver disease, n (%)	25 (6.87)	17 (14.7)	8 (3.23)	<0.001
Diabetes, n (%)	88 (24.2)	21 (18.1)	67 (27.0)	0.063
Renal disease, n (%)	30 (8.24)	9 (7.76)	21 (8.47)	0.837
Malignant cancer, n (%)	105 (28.8)	38 (32.8)	67 (27.0)	0.264
**CCI, Mean (SD)**	4.80 (3.15)	4.79 (3.49)	4.80 (2.98)	0.987
**Primary Infection source**				
Lung, n (%)	148 (40.7)	43 (37.1)	105 (42.3)	0.401
Abdomen, n (%)	52 (14.3)	25 (21.6)	27 (10.9)	0.011
Urinary, n (%)	34 (9.3)	8 (6.9)	26 (10.5)	0.367
Skin and soft tissue, n (%)	13 (3.6)	8 (6.9)	5 (2.0)	0.030
Central nervous system, n (%)	6 (1.6)	2 (1.7)	4 (1.6)	1.000
Endocardium, n (%)	2 (0.6)	1 (0.9)	1 (0.4)	0.536
Intravascular devices, n (%)	50 (13.7)	16 (13.8)	34 (13.7)	1.000
other, n (%)	7 (1.92)	0 (0.0)	7 (2.8)	0.102
Unknown, n (%)	105 (28.8)	35 (30.2)	70 (28.2)	0.797
**Lab value, median [IQR]**				
WBC, ×10^9^/L	12.2 [7.54;17.6]	10.7 [3.43;16.7]	12.6 [8.85;17.9]	0.001
PT, second	15.4[13.2;17.6]	15.4[13.9;15.4]	14.4[12.9;16.9]	<0.001
APTT, second	34.8[29.0;44.6]	41.6 [33.0;48.6]	32.0[28.3;40.8]	<0.001
Bilirubin, mg/dl	0.9 [0.5; 2.0]	1.6 [0.8; 3.4]	0.8 [0.5; 1.4]	<0.001
Creatinine, mg/dl	0.9 [0.6; 1.5]	1.0 [0.7;1.9]	0.8 [0.6;1.4]	0.005
**Microorganism**				0.025
*Enterococcus* species, n (%)	48 (13.19)	10 (8.62)	38 (15.3)	
*Enterobacter* species, n (%)	17 (4.67)	2 (1.72)	15 (6.05)	
*Staphylococcus* species, n (%)	42 (11.54)	12 (10.3)	30 (12.1)	
*Candida* species, n (%)	64 (17.58)	23 (19.8)	41 (16.5)	
*Escherichia* species, n (%)	94 (25.82)	30 (25.9)	64 (25.8)	
*Klebsiella* species, n (%)	53 (14.56)	24 (20.7)	29 (11.7)	
*Pseudomonas* species, n (%)	33 (9.06)	10 (8.62)	23 (9.27)	
*Burkholderia* species, n (%)	8 (2.20)	1 (0.86)	7 (2.82)	
*Serratia* species, n (%)	5 (1.37)	4 (3.45)	1 (0.40)	
**APACHE II, median [IQR]**	16 [11; 23]	19 [14; 26]	15 [9; 22]	<0.001
**SOFA score, median [IQR]**	5 [3; 9]	9 [6; 13]	4 [2; 7]	<0.001
**Anti-platelet drugs, n (%)**	69 (19.0)	17 (14.7)	52 (21.0)	0.152
Septic Shock, n (%)	151 (41.5)	68 (58.6)	83 (33.5)	<0.001
Vasopressor usage, n (%)	100 (27.5)	51 (44.0)	49 (19.8)	<0.001
Mechanical Ventilation usage, n (%)	128 (35.2)	57 (49.1)	71 (28.6)	<0.001
Extra-corporeal epuration, n (%)	168 (39.3)	104 (39.4)	64 (40.7)	0.717
**Mortality**				
28-day death, n (%)	73 (20.1)	36 (31.0)	37 (14.9)	0.001
In hospital death, n (%)	54 (14.8)	29 (25.0)	25 (10.1)	<0.001

Date expressed as mean (SD) or median(Q1-Q3) or number (percentage). *PLT*, Platelet; *CCI*, Charlson comorbidity index; *WBC*, White blood cells; *PT*, prothrombin time; *APTT*, activated partial thromboplatin time; *APACHE*, Acute Physiology and Chronic Health Evaluation; *SOFA*, Sequential Organ Failure Assessment score.

In both cohorts, patients with thrombocytopenia were younger, and more likely to have liver disease and malignant cancer. Patients without thrombocytopenia were older, and more likely to have diabetes (Tables 1 and 2).

### Association between specific pathogens and thrombocytopenia in the MIMIC cohort

There were significant differences in the circulating nadir platelet counts (*p* < 0.001) and the proportion of patients with thrombocytopenia for different pathogens (*p* = 0.003) (eTable S1 and eFigure S1a in Supplementary material 1). The incidence of thrombocytopenia among patients was 37.5% in those with *Candida* infections, followed by 35.4% in *Serratia* infections, 34.1% in *Enterobacter* infections, 30.8% in *Escherichia* infections, and 29.3% in *Klebsiella* infections.

**Figure 2.**
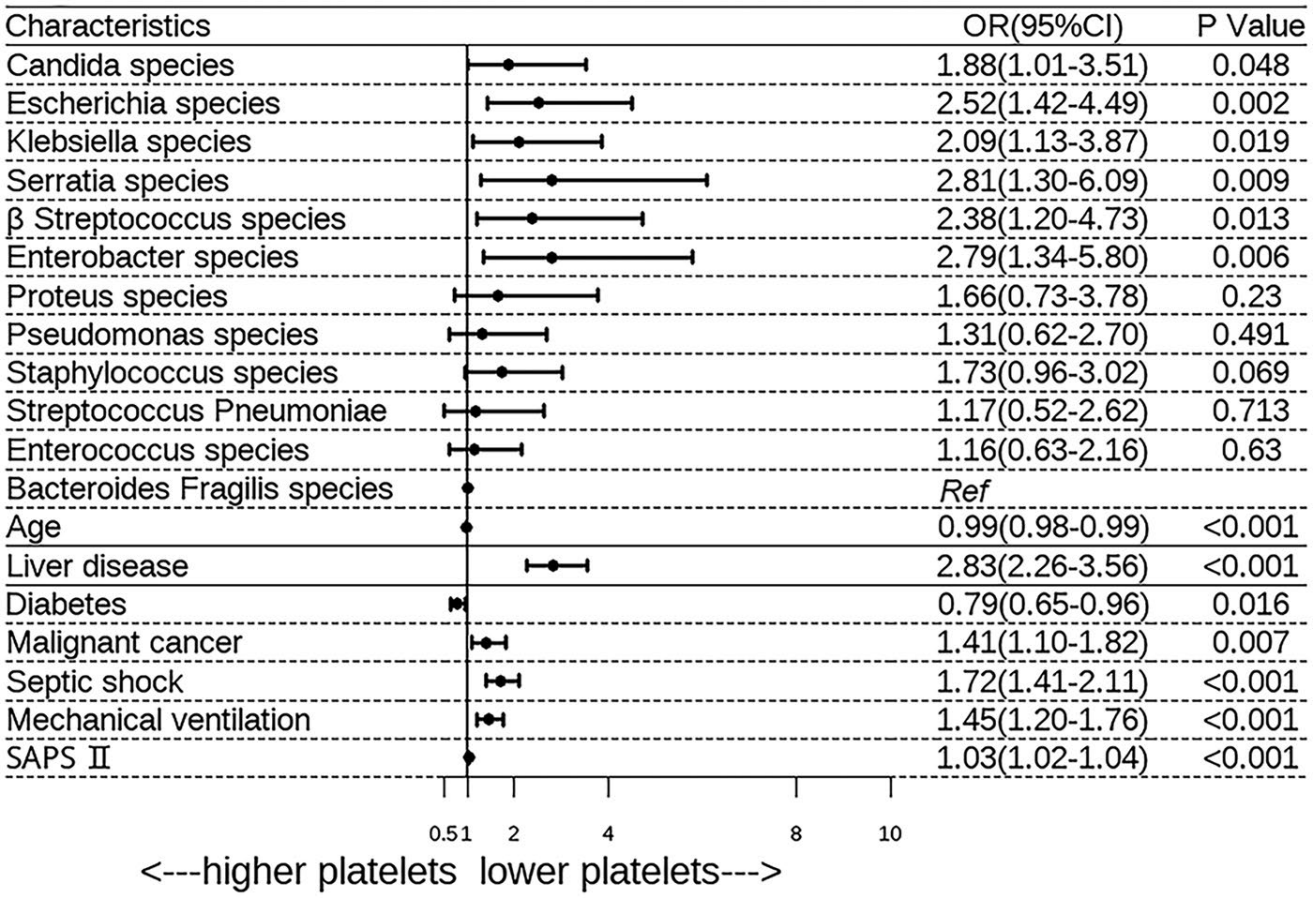
Multivariable adjusted association of specific pathogen with incidence of thrombocytopenia in MIMIC cohort. Additional potential confounders added by stepwise selection. *SAPS*, Simplified Acute Physiology Score; *CI*, confidence interval; *OR*, odds ratio.

The risk factors identified through univariate analysis are presented in Table 1. There was a significant difference in the distribution of pathogens between the two groups (*p* = 0.003). Then we evaluated the risk of thrombocytopenia using the multivariable logistic regression model adjusted for age, comorbidities, septic shock, SAPS II score, mechanical ventilation use, and antiplatelet drug use (Figure 2). After adjusting for potential confounders, we found that *Candida* species (OR, 1.88; 95% CI, 1.01 − 3.51), *Enterobacter* species (OR, 2.79; 95% CI, 1.34 − 5.80), *Escherichia* species (OR, 2.52; 95% CI, 1.42 − 4.49), *Klebsiella* species (OR, 2.09; 95% CI, 1.13 − 3.87), *Serratia* species (OR, 2.81; 95% CI, 1.30 − 6.09), and *β Streptococcus* species (OR, 2.38; 95% CI, 1.20 − 4.73) had a significantly higher risk of thrombocytopenia.

As thrombocytopenia during the infectious period may be caused by other factors, the risk of thrombocytopenia according to pathogen species was further determined using PSM populations. Patient characteristics of the PSM populations are shown in eTable S2 in Supplementary material 2. Multivariate logistic regression analysis confirmed that *Candida*, *Escherichia*, *Klebsiella*, *Enterobacter*, *β Streptococcus*, and *Serratia* species had a higher risk of thrombocytopenia than other pathogen species (Table 3).

**Table 3.** Multivariable analysis for thrombocytopenia using PSM population of MIMIC cohort.

	OR	95% CI	*p*
*Bacteroides Fragilis* species	1.00	*Ref*	*Ref*
*Escherichia* species*^a^*	2.65	1.37-5.39	0.005
*Klebsiella* species	2.29	1.05-5.14	0.040
*Candida* species	2.01	1.02-4.09	0.047
*Enterobacter* species	2.29	1.05-5.14	0.040
*Serratia* species*^b^*	3.84	1.52-10.24	0.005
*β Streptococcus* species*^c^*	3.01	1.39-6.78	0.006

Multivariable Logistic regression model for influence of pathogens on platelet response in PSM population, additional potential confounders (including age, comorbidities, septic shock, SAPS II score, and Mechanical ventilation use and antiplatelet drugs use) added by stepwise backward selection. *a*. variables as mild liver disease, septic shock, SAPS II were also significant in the model. *b.* variables as septic shock, SAPS II were also significant in the model. *c.* variables as mild liver disease, septic shock, SAPS II were also significant in the model. *SAPS* Simplified Acute Physiology Score, *CI* confidence interval, *OR*: odds ratio.

**Figure 3.**
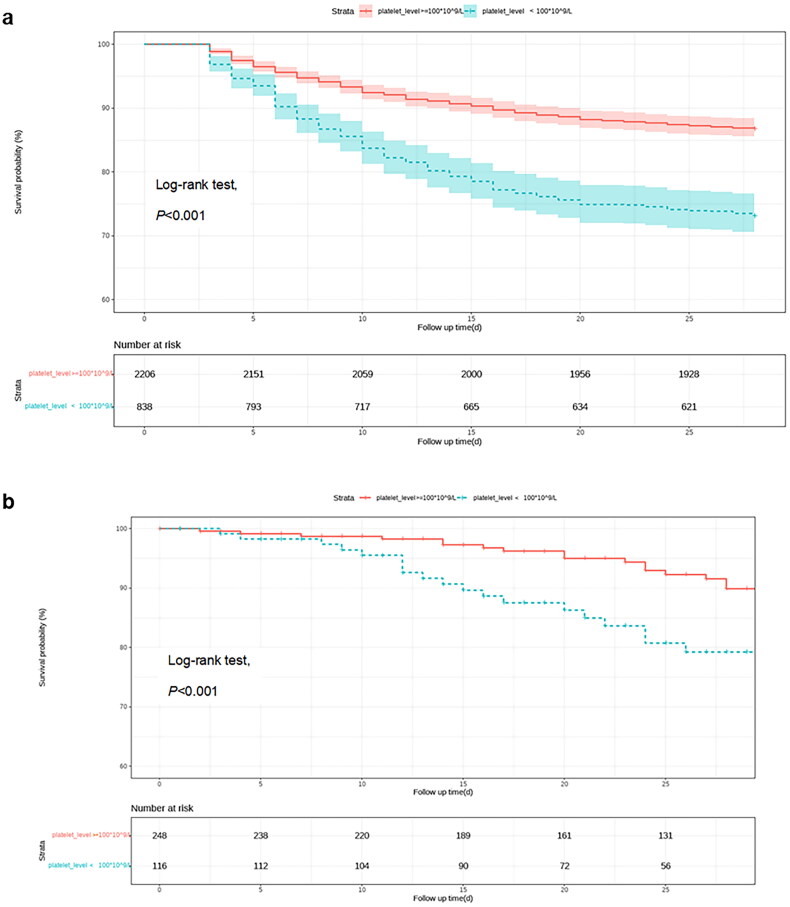
Kaplan–Meier analysis estimates 28d survival of septic patients with or without thrombocytopenia. (a) MIMIC cohort; (b) SBISC cohort.

**Figure 4.**
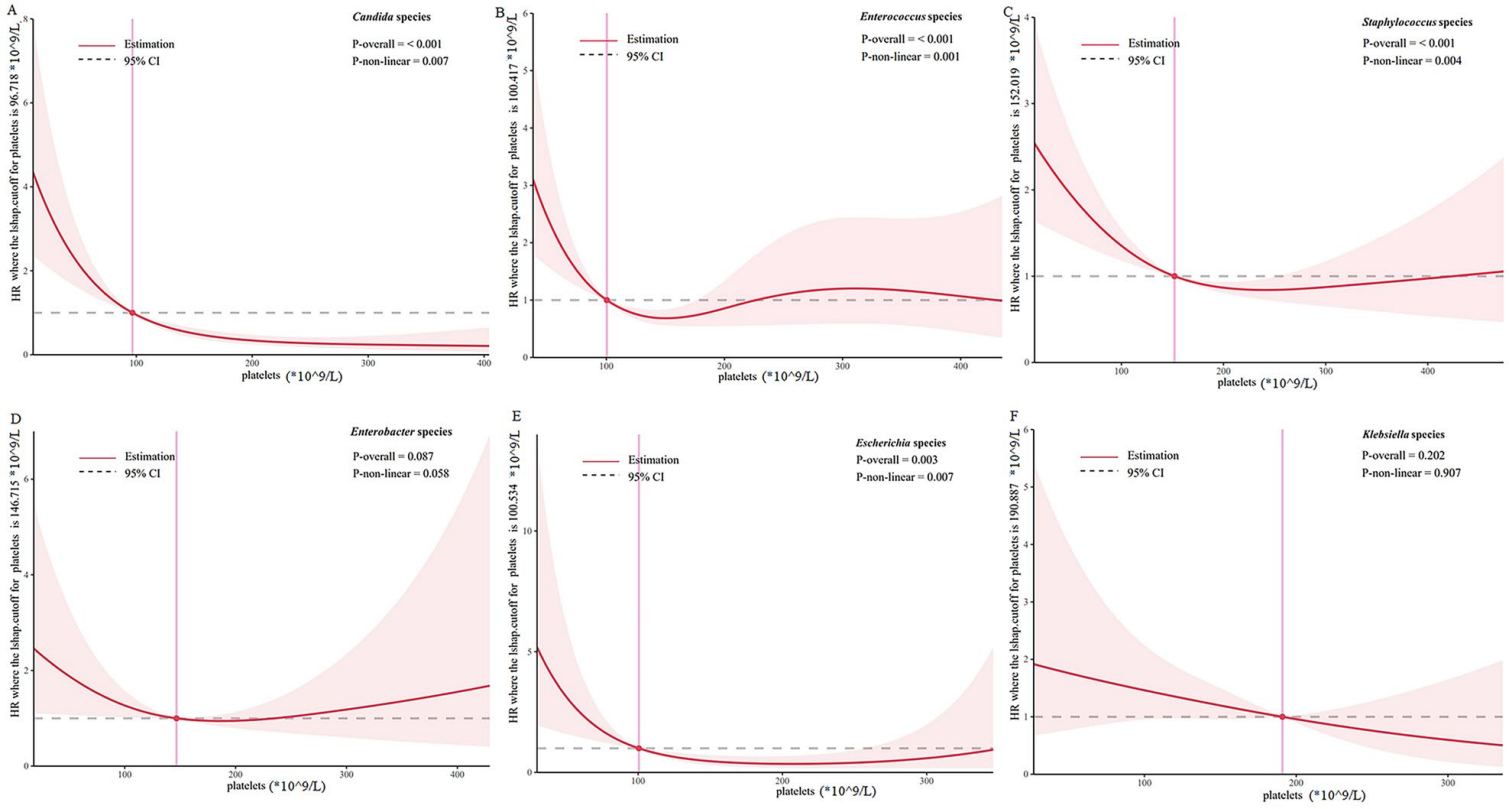
The nonlinear association between platelet counts and 28d all-cause mortality by restricted cubic spline models in specific pathogens. (A) *Candida* species. (B) *Enterococcus* species. (C) *Staphylococcus* species. (D) *Enterobacter* species. (E) *Escherichia* species. (F) *Klebsiella* species. The models were adjusted by age, gender, medical conditions, Simplified Acute Physiology Score (SAPS) II score, septic shock, mechanical ventilation use and lactate. All *p* values were corrected for multiple testing by using the likelihood ratio method. *HR*, hazard ratio; *CI*, confidence intervals. The L-shaped relationship indicated a decrease in the hazard ratio for mortality with increasing platelet count, characterized by steeper slopes at lower counts and tending to stabilize at higher counts. Conversely, the U-shaped relationship indicates a decrease in the hazard ratio for mortality with increasing platelet count, followed by an increase as the count further increases.

In order to discover the relationship between platelet count and 28-day all-cause mortality, we first analysed the association between thrombocytopenia and 28-day all-cause mortality in all patients using Cox proportional hazard models. Multivariate Cox regression analysis should that thrombocytopenia was an independent risk factor for 28-day mortality (HR = 1.56, 95% CI = 1.31-1.87, *p* < 0.001; Table 4). Kaplan-Meier analysis showed that compared with patients without thrombocytopenia, patients with thrombocytopenia had a significant increased risk of 28-day mortality (log-rank *p* < 0.001, Figure 3a). We further used restricted cubic spline (RCS) based on multivariable Cox proportional hazard models to analyse the relationships between circulating platelet count and 28d all-cause mortality in specific pathogens in MIMIC cohort. After adjusting for confounders, RCS analysis showed an L- or U-shaped relationship in *Candida* species, *Enterococcus* species, *Escherichia* species, *Enterobacter* species, *Staphylococcus* species and *Klebsiella* species, at platelet count cutoff values of 96 × 10^9^/L, 100 × 10^9^/L, 100 × 10^9^/L, 146 × 10^9^/L, 152 × 10^9^/L, and 190 × 10^9^/L, respectively (Figure 4).

**Table 4.** Univariable and multivariable association of thrombocytopenia with 28d all-cause mortality in all patients.

MIMIC cohort			Univariable models		Multivariable models	
outcome	Platelet Level	n	HR (95% CI)	*p*	HR (95% CI)	*P* ^1^
Death from all cause	**PLT> =100 × 10^9^/L**	2206	1.00 (reference)		1.00 (reference)	
	**PLT < 100 × 10^9^/L**	838	2.20 (1.85-2.62)	<0.001	1.56 (1.31-1.87)	<0.001
SBISC cohort			Univariable models		Multivariable models	
outcome	Platelet Level	n	HR (95% CI)	*P*	HR (95% CI)	*P* ^2^
Death from all cause	**PLT> =100 × 10^9^/L**	248	1.00 (reference)		1.00 (reference)	
	**PLT < 100 × 10^9^/L**	116	2.59 (1.64-4.11)	<0.001	1.76 (1.05-2.77)	0.030

Cox regression model for influence of thrombocytopenia on 28d all-cause mortality, additional potential confounders added by stepwise selection.^1^The adjusted variables were age, gender, comorbidities, SAPS II score, septic shock, management, including vasopressor and mechenical ventilation. ^2^The adjusted variables were age, gender, comorbidities, APACHE II score, septic shock, management, including vasopressor and mechenical ventilation. *PLT*, platelet; *CI*, confidence interval; *HR*, hazard ratio.

### Association between specific pathogens and thrombocytopenia in the SBISC cohort

As observed in the MIMIC cohort, there were significant differences in circulating nadir platelet counts (*p* = 0.017) and the proportion of patients with thrombocytopenia for different pathogens (*p* = 0.025) (eTable S1 and eFigure S1b in Supplementary material 1).

**Figure 5.**
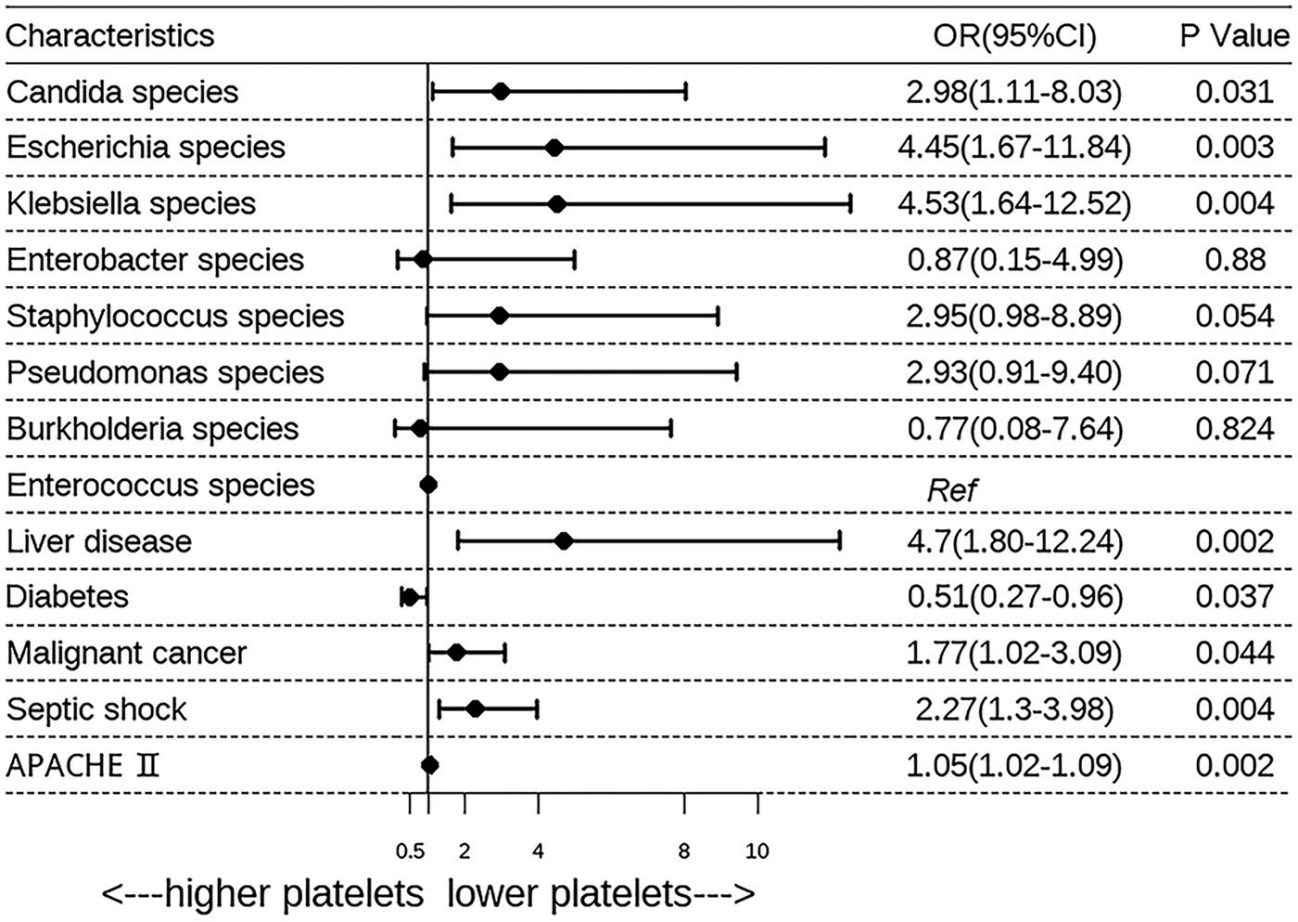
The multiple analysis for association between specific pathogens and incidence of thrombocytopenia in SBISC cohort. Additional potential confounders added by stepwise selection. *APACHE*, Acute Physiology and Chronic Health Evaluation; *CI*, confidence interval; *OR*, odds ratio.

The risk factors identified through univariate analysis are presented in Table 2. There was a significant difference in the distribution of pathogens between the two groups (*p* = 0.025). As observed in the MIMIC cohort, after adjustment for potential confounders, a multiple logistic regression model showed that thrombocytopenia was more common in patients with sepsis caused by *Candida* species (OR, 2.98; 95% CI, 1.11 − 8.03), *Escherichia* species (OR, 4.45; 95% CI, 1.67 − 11.84), *Klebsiella* species (OR, 4.53; 95% CI, 1.64 − 12.52), and *Serratia* species (OR, 49.65; *p* = 0.002), while it was least common in patients infected with *Staphylococcus*, *Enterococcus*, and *Pseudomonas* species (Figure 5). As there was only a small number of *Serratia* species (*n* = 5), the OR for thrombocytopenia was not accurate enough. Additionally, *Enterobacter* species were not identified as common pathogens associated with thrombocytopenia, and this may be explained by the small number of patients with infections caused by *Enterobacter* species in this cohort. Thrombocytopenia was also identified as an independent risk factor for 28-day mortality in this cohort (HR = 2.3, 95% CI = 1.28 − 4.16, *p* = 0.030; Table 4), and the 28-day survival curve of patients with or without thrombocytopenia is presented in Figure 3b.

## Discussion

The main finding of this study is the pathogen-specific platelet changes in two large cohorts, encompassing a broad range of microbial species. The results indicate that patients with sepsis caused by *Candida*, *Escherichia*, *Klebsiella*, and *Serratia* species is more frequently associated with thrombocytopenia. We observed L- or U-shaped associations with different cutoff values between platelet counts and 28d-mortality in *Candida*, *Enterococcus*, *Escherichia*, *Enterobacter*, *Staphylococcus*, and *Klebsiella* species. Our findings suggest that clinicians should pay particular attention to the potential thrombocytopenia in patients infected with the above microorganisms, as thrombocytopenia is a potential risk factor for poor prognosis.

Platelets play a crucial role in host immune response against infection, and diminished platelet counts often indicate a disruption in host response mechanisms [6]. The occurrence of thrombocytopenia, which is defined as a circulating platelet count <100 × 10^9^/L, correlates strongly with increased mortality rates [16]. The underlying mechanisms of infection-induced thrombocytopenia are complex, and the most common causative mechanisms are platelet consumption and immune thrombocytopenia triggered by cell-specific membrane components of microorganisms, such as endotoxins, lipopolysaccharides, and bacterial exotoxins. Multiple complicated mechanisms of infection-associated thrombocytopenia have been detected in several pathogens [1, 10, 17–21]. However, it is not clear whether the risk of thrombocytopenia varies among different pathogens. Our study is the largest, to date, to evaluate the changes of platelet count and to determine the association between platelet counts and the prognosis in specific pathogens.

Fungi and bacteria can interact with platelets, triggering activation and aggregation [7]. This interaction occurs either directly *via* receptors on the platelets or indirectly through molecular intermediaries like fibrinogen [5, 19]. As a result, there are differences between pathogens in terms of their ability to induce platelet activation and aggregation [21], which can lead to thrombocytopenia owing to amplification of the activation process. Accordingly, the findings of the present study documents variations in platelet count and the prevalence of thrombocytopenia among different pathogens.

In our study, both in the MIMIC and SBISC cohorts, *Candida, Escherichia, Klebsiella*, and *Serratia* species were the most common pathogens associated with thrombocytopenia, while *Staphylococcus*, *Enterococcus*, and *Pseudomonas* species were the least common thrombocytopenia-associated pathogens. This variation in thrombocytopenia incidence among different pathogens aligns with previous studies in neonate and adult patient populations. A retrospective study with 214 septic patients admitted to the ICU reported that there was a difference in platelet response to different types of organisms: patients with fungal sepsis had a lower platelet count and a longer duration of thrombocytopenia than those with sepsis caused by Gram-negative and Gram-positive bacterial species [22]. In a similar 4-year investigation on 154 very-low-birth-weight neonates with sepsis admitted to the neonatal ICU, patients caused by Gram-negative bacterial species or fungi had a significantly lower platelet count and higher incidence of thrombocytopenia than those with sepsis caused by Gram-positive bacterial species [23]. However, these two investigations included only a small number of cases and analysed the differences in platelet response to different classes of organisms but not to specific pathogens. On the contrary, another retrospective study involving 600 patients with bacteriaemia showed no significant difference in the incidence of thrombocytopenia between patients with bacteriaemia caused by *S. aureus* and those caused by *E. coli* and *S. pneumoniae* [24]. This previous study also differs from our study because it focused specifically on bacteriaemia caused by *E. coli* and *S. pneumoniae* as one group.

During episodes of sepsis, thrombocytopenia often results from microvascular thrombosis and direct platelet consumption. Consequently, it is worth considering whether the early administration of drugs that inhibit platelet aggregation, in conjunction with precise antimicrobial treatments, might benefit septic patients. Moreover, considering the pathogen-specific responses of platelet counts and the corresponding thresholds associated with sepsis-related mortality, proactive measures to elevate platelet counts within normal limits should be considered to mitigate thrombocytopenia risks.

Our study has several limitations. First, it was an observational study, so it was difficult to account for all confounders that might influence the effect of pathogens on platelet count, even after adjusting for confounders by PSM and multivariable Cox regression. However, we have excluded patients with exposure to several well-known risk factors, including pre-existing thrombocytopenia, severe liver disease, AIDS, as well as radiotherapy or chemotherapy within the preceding 3 months, to minimize the possible effect of confounders. Second, the evaluation of treatment effects on platelet count remains inconclusive. Although we excluded the presence of other conditions that could cause thrombocytopenia, such as drug-induced effects, at baseline, the potential impact of treatment, particularly certain antibiotics like zosyn and linezolid, on platelet count may still exist. To minimize this potential effect, we conducted an analysis of platelet counts recorded within the initial two days following sepsis onset. Third, our local prospective cohort was a small one, so the number of pathogen species and number of patients were limited. Forth, as the occurrence of DIC following sepsis is a complex manifestation of severe infection, our study did not further investigate this complication. Future prospective research involving more centers and more cases could further explore the pathogen-specific effects on platelet count in more detail.

## Conclusion

In conclusion, we have provided evidence for a pathogen-specific changes on platelet count and L- or U-shaped associations with different cutoff values between platelet count and 28d-mortality in septic patients with various pathogen infections. Specifically, patients infected with *Candida, Escherichia*, *Klebsiella*, and *Serratia* species were found to be at a higher risk for thrombocytopenia. Potential thrombocytopenia is especially worth monitoring in patients infected with the above microorganisms. Furthermore, early interventions on platelet at various threshold should be considered in septic patients infected with diverse pathogens.

## Supplementary Material

Supplemental Material

## Data Availability

The MIMIC datasets used and/or analysed during the current study are available in [MIMIC database] at https://mimic.physionet.org/. reference number [11,12]. Researchers are required to complete a collaborative institution training initiative program course in order to access the database. The SBISC datasets used and/or analysed during the current study are available from the corresponding author on reasonable request.
