# Supplementary material for: Quantitative changes in platelet count in response to different pathogens: an analysis of patients with sepsis in both retrospective and prospective cohorts
Source: Ann Med. 2024 Sep 20;56(1):2405073. doi: 10.1080/07853890.2024.2405073 (PMC11418053; doi:10.1080/07853890.2024.2405073)
Supplement: Supplemental Material [file IANN_A_2405073_SM7131.zip › Supplementary material 1 & 2/Supplementary material 2.docx]

**Supplementary Materials 2**

**The supplementary material includes a checklist, 1 supplementary table.**

**Checklist:**

**eTable S2:** Patients’ characteristic in the propensity matched populations of MIMIC.

**eTable S2.** Patients’ characteristic in the propensity matched populations of MIMIC

| Variables | *Bacteroides Fragilis* species n=86 | *Escherichia* species n=146 | *p1* | *Bacteroides Fragilis* species n=86 | *Klebsiella* species n=104 | *p2* | *Bacteroides Fragilis* species n=87 | *Candida* species n=104 | *p3* |
| --- | --- | --- | --- | --- | --- | --- | --- | --- | --- |
| Age, Mean (SD) | 69.8 (13.8) | 68.8(14.8) | 0.591 | 69.8 (13.9) | 69.7(15.0) | 0.981 | 69.8 (13.6) | 67.3 (15.2) | 0.222 |
| **Gender** |  |  | 0.100 |  |  | 0.974 |  |  | 0.363 |
| Male, n (%) | 51 (58.6) | 269 (50.0) |  | 51 (60.0) | 61(58.7) |  | 51 (58.6) | 53 (51.0) |  |
| Female, n (%) | 35 (40.7) | 77 (52.7) |  | 34 (40.0) | 43 (41.3) |  | 36 (41.4) | 51 (49.0) |  |
| **Medical conditions** |  |  |  |  |  |  |  |  |  |
| Myocardial infarct, n (%) | 13 (15.1) | 20 (13.7) | 0.920 | 13 (15.3) | 16 (15.4) | 1.000 | 13 (14.9) | 13 (12.5) | 0.780 |
| Congestive heart failure, n (%) | 24 (27.9) | 29 (19.9) | 0.210 | 23 (27.1) | 29 (27.9) | 1.000 | 24 (27.6) | 39 (37.5) | 0.200 |
| Chronic pulmonary disease, n (%) | 35 (40.7) | 32 (21.9) | 0.004 | 35 (41.2) | 19 (18.3) | 0.001 | 36 (41.4) | 30 (28.8) | 0.100 |
| Mild liver disease, n (%) | 13 (15.1) | 25 (17.1) | 0.832 | 12 (14.1) | 18 (17.3) | 0.690 | 15 (17.2) | 18(17.3) | 1.000 |
| Diabetes, n (%) | 18 (20.9) | 30 (20.5) | 1.000 | 18 (21.2) | 32 (30.8) | 0.193 | 18 (20.7) | 25 (24.0) | 0.712 |
| Renal disease, n (%) | 7 (8.14) | 22 (15.1) | 0.187 | 7 (8.24) | 19 (18.3) | 0.082 | 9 (10.3) | 13 (12.5) | 0.811 |
| Malignant cancer, n (%) | 16 (18.6) | 34 (23.3) | 0.500 | 16 (18.8) | 19 (18.3) | 1.000 | 16 (18.4) | 16 (15.4) | 0.723 |
| **Antiplatelet drugs, n (%)** | 23 (26.7) | 38 (26.0) | 1.000 | 22 (25.9) | 20 (19.2) | 0.361 | 23 (26.4) | 28 (26.9) | 1.000 |
| **SAPS II, Mean (SD)** | 42 (13.3) | 40 (12.1) | 0.162 | 42(13.4) | 41(13.6) | 0.742 | 43(14.7) | 44(14.4) | 0.780 |
| Septic Shock, n (%) | 63 (73.3) | 104 (71.2) | 0.861 | 63 (72.9) | 75 (72.1) | 1.000 | 64 (73.6) | 81 (77.9) | 0.601 |
| Mechanical Ventilation usage, n (%) | 39 (45.3) | 61 (41.8) | 0.690 | 39 (44.7) | 40 (38.5) | 0.473 | 41(47.1) | 57 (54.8) | 0.362 |

| Variables | *Bacteroides Fragilis* species n=87 | *β Streptococcus* species n=146 | *p4* | *Bacteroides Fragilis* species n=86 | *Serratia* species n=49 | *p5* | *Bacteroides Fragilis* species  n=85 | *Enterobacter* species n=62 | *p6* |
| --- | --- | --- | --- | --- | --- | --- | --- | --- | --- |
| Age, Mean (SD) | 70 (13.8) | 64.8(18.8) | 0.048 | 70 (13.7) | 63.7 (14.8) | 0.014 | 69.7 (13.8) | 66.8 (13.5) | 0.201 |
| **Gender** |  |  | 0.970 |  |  | 1.000 |  |  | 0.952 |
| Male, n (%) | 51 (58.6) | 44 (57.1) |  | 51 (58.0) | 29 (59.2) |  | 51 (60.0) | 36 (58.1) |  |
| Female, n (%) | 36 (41.4) | 33 (42.9) |  | 37 (42.0) | 20 (40.8) |  | 34 (40.0) | 26 (41.9) |  |
| **Medical conditions** |  |  |  |  |  |  |  |  |  |
| Myocardial infarct, n (%) | 13 (14.9) | 10 (13.0) | 0.890 | 13 (14.8) | 9 (18.4) | 0.760 | 13 (15.3) | 14 (22.6) | 0.360 |
| Congestive heart failure, n (%) | 24 (27.6) | 25 (32.5) | 0.612 | 24 (27.3) | 14 (28.6) | 1.000 | 24 (28.2) | 15 (24.2) | 0.723 |
| Chronic pulmonary disease, n (%) | 36 (41.4) | 14 (18.2) | 0.002 | 37(42.0) | 17 (34.7) | 0.511 | 34 (40.0) | 14 (22.6) | 0.040 |
| Mild liver disease, n (%) | 14 (16.1) | 10 (13.0) | 0.733 | 15 (17.0) | 9 (18.4) | 1.000 | 12 (14.1) | 6 (9.7) | 0.581 |
| Diabetes, n (%) | 18 (20.7) | 23 (29.9) | 0.242 | 18 (20.5) | 18 (36.7) | 0.061 | 18 (21.2) | 15 (24.2) | 0.822 |
| Renal disease, n (%) | 9 (10.3) | 12 (15.6) | 0.443 | 9 (10.2) | 8 (16.3) | 0.443 | 7 (8.24) | 8 (12.9) | 0.522 |
| Malignant cancer, n (%) | 15(17.2) | 8 (10.4) | 0.305 | 16 (18.2) | 4 (8.16) | 0.182 | 15(17.6) | 4 (6.5) | 0.081 |
| **Antiplatelet drugs, n (%)** | 23 (26.4) | 22 (28.6) | 0.908 | 23 (26.1) | 20 (40.8) | 0.113 | 23 (27.1) | 22 (35.5) | 0.361 |
| **SAPS II, Mean (SD)** | 42 (14.4) | 38 (13.0) | 0.021 | 43 (14.6) | 38 (12.8) | 0.042 | 42 (12.9) | 39 (12.8) | 0.234 |
| Septic Shock, n (%) | 64 (73.6) | 48 (62.3) | 0.173 | 65 (73.9) | 37 (75.5) | 0.991 | 62 (72.9) | 40 (64.5) | 0.360 |
| Mechanical Ventilation usage, n (%) | 40 (46.0) | 31 (40.3) | 0.560 | 41 (46.6) | 26 (53.1) | 0.580 | 38(44.7) | 32 (51.6) | 0.510 |

Date expressed as mean (sd) or number (percentage). *IQR* interquartile range, *SAPS* Simplified Acute Physiology Score.
