# Supplementary material for: Quantitative changes in platelet count in response to different pathogens: an analysis of patients with sepsis in both retrospective and prospective cohorts
Source: Ann Med. 2024 Sep 20;56(1):2405073. doi: 10.1080/07853890.2024.2405073 (PMC11418053; doi:10.1080/07853890.2024.2405073)
Supplement: Supplemental Material [file IANN_A_2405073_SM7131.zip › Supplementary material 1 & 2/Supplementary material 1.docx]

**Supplementary Materials 1**

**The supplementary material includes a checklist, eMethods, 1 supplementary table (eTable S1), and 1 supplementary figure (eFigure S1).**

**Checklist:**

**eMethods**

**eTable S1:** The nadir platelet counts among specific pathogens.

**eFigure S1:** The distribution of the proportion of sepsis patients with thrombocytopenia among different pathogens.

**eMethods**

***Variables***

Variables with missing data are common in the MIMIC database. In cases with <20% missing data, the missing data were multiple imputed using the random forest method (mice package for R). Our SBISC cohort has a small amount of missing data, and the laboratory values of total bilirubin and creatinine had the highest rate of missing data, as they were missing in 8% and 5% of patients, respectively. Since both of these are continuous variables, we used the predictive mean matching method to impute predictions.

***Definitions***

The day of sepsis onset was defined as the day on which the first positive blood culture specimen was collected. Mechanical ventilation was defined as the use of invasive and non-invasive mechanical ventilation interventions. 28-day mortality was defined as all-cause death within 28 days after sepsis onset.

***Statistical analysis***

As thrombocytopenia during the infectious period may be caused by other factors, we used propensity score matching (PSM) to reduce mismatch among groups infected by different pathogens and determine the association between specific pathogens and thrombocytopenia. We calculated propensity scores by using a logistic regression model that included all variables associated with baseline and disease severity that were different between pathogen groups (R library MatchIt). We matched subjects in different groups of pathogens using a nearest neighbor matching method with a caliper of 0.2OD and a suitable match ratio (1:1 or 1:2). Standard mean differences were calculated to evaluate whether the groups being compared were matched.

**eTable S1.** The nadir platelet counts among specific pathogens

|  | Number | PLT, ×10^9^/L, median (IQR) | *p* |
| --- | --- | --- | --- |
| Nadir platelet count in MIMIC cohort | | | |
| **Microorganism** |  |  | <0.001 |
| *Bacteroides Fragilis* species | 88 | 221 (111,288) |  |
| *β Streptococcus* species | 135 | 139 (92,187) |  |
| *Candida* species | 220 | 134 (76,235) |  |
| *Enterobacter* species | 81 | 138 (79,200) |  |
| *Enterococcus* species | 318 | 175 (115,247) |  |
| *Escherichia* species | 728 | 134 (86,199) |  |
| *Klebsiella* species | 273 | 142 (92,198) |  |
| *Proteus* species | 61 | 126 (102,170) |  |
| *Pseudomonas* species | 92 | 159 (96,243) |  |
| *Serratia* species | 65 | 141 (58,193) |  |
| *Staphylococcus* species | 906 | 159 (99,229) |  |
| *Streptococcus Pneumoniae* | 77 | 165 (123,228) |  |
|  | Number | PLT, ×10^9^/L, median (IQR) | *p* |
| Nadir platelet count in SBISC cohort | | | |
| **Microorganism** |  |  | 0.017 |
| *Enterococcus* species | 48 | 195 (126,307) |  |
| *Enterobacter* species | 17 | 179 (138, 274) |  |
| *Staphylococcus* species | 42 | 162 (87,250) |  |
| *Candida* species | 64 | 149 (65,225) |  |
| *Escherichia* species | 94 | 146 (63,236) |  |
| *Klebsiella* species | 53 | 134 (42,244) |  |
| *Pseudomonas* species | 33 | 176 (71,218) |  |
| *Burkholderia* species | 8 | 319 (175,456) |  |
| *Serratia* species | 5 | 75 (60,97) |  |

Absolute numbers of microorganisms. *p* value for difference among pathogens. *IQR* interquartile range*, PLT* platelet.


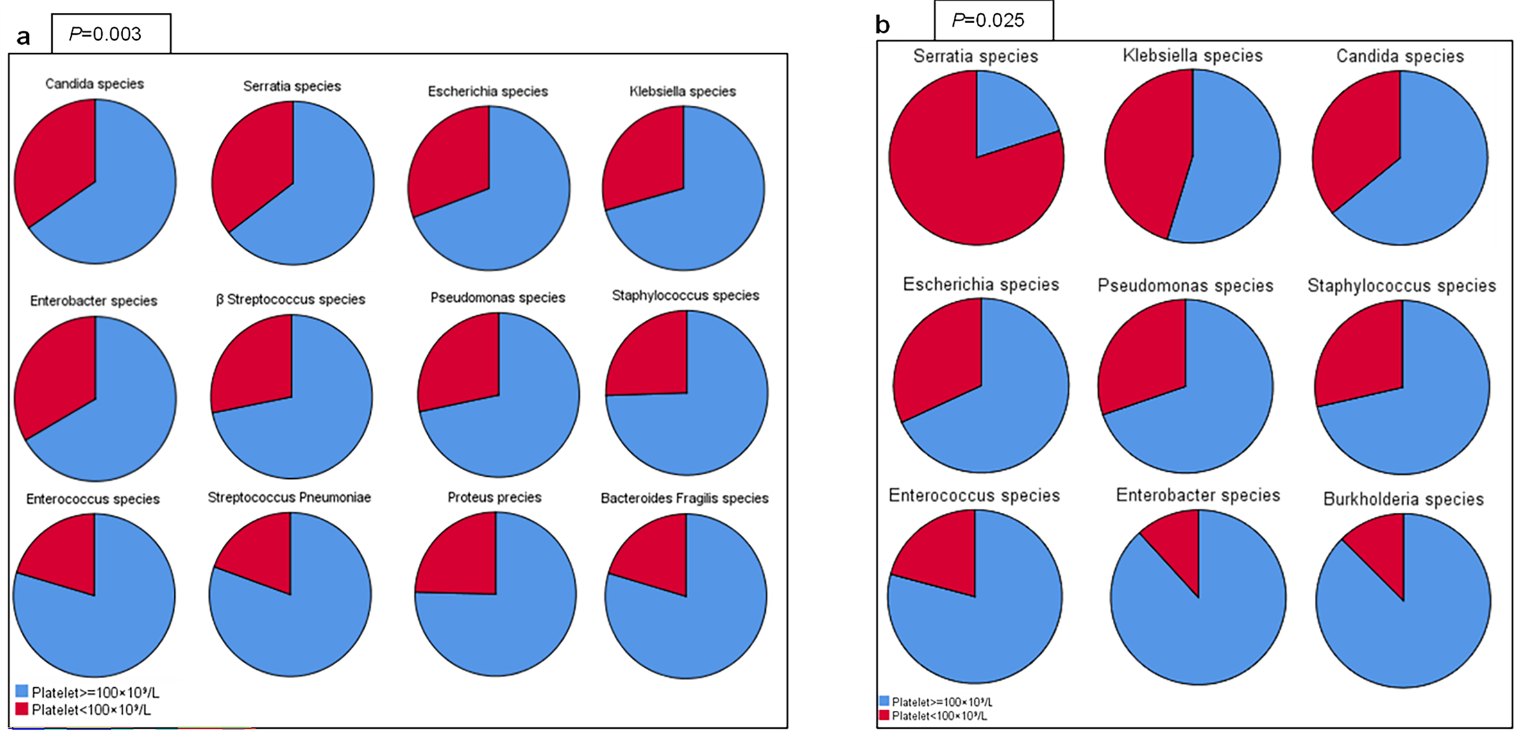


**eFigure S1. The distribution of the proportion of sepsis patients with thrombocytopenia among different pathogens.** (a) MIMIC cohort; (b) SBISC cohort. *p* value for difference among pathogens.
